# Identify Diagnostic Biomarkers Related to Taurine Metabolism in Diabetic Foot Ulcers Using Bulk RNA‐seq and ScRNA‐seq Analysis

**DOI:** 10.1111/1753-0407.70166

**Published:** 2026-01-08

**Authors:** Mengrong He, Jiamin Chen

**Affiliations:** ^1^ Hand Surgery/Wound Repair Surgery/Burns Department Lishui Hospital of Wenzhou Medical University, The First Affiliated Hospital of Lishui University, Lishui People's Hospital Lishui China

**Keywords:** diabetic foot ulcers, diagnostic biomarkers, immune response, scRNA‐seq, taurine metabolism

## Abstract

**Background:**

Diabetic foot ulcers (DFU) are severe complications with complex pathogenesis involving inflammation and impaired healing. Taurine, a key antioxidant amino acid, shows therapeutic potential in diabetes, but its role in DFU remains unclear and warrants investigation.

**Methods:**

This integrated multi‐omics study analyzed DFU using GEO transcriptomics data (training: GSE68183/GSE80178; validation: GSE37265/GSE134431; scRNA‐seq: GSE223964). Limma identified DEGs that intersected with taurine metabolism ‐related genes. Key modules (MCODE) and a LASSO‐based diagnostic signature were established. Immune infiltration was profiled via ssGSEA, CIBERSORT, and MCP‐Counter. Regulatory networks (TFs/miRNAs) were predicted (miRNet/NetworkAnalyst), and therapeutic agents were screened (DSigDB). Seurat‐processed scRNA‐seq defined nine cell types, with CellChat analyzing intercellular communication.

**Results:**

This study identified a three‐gene diagnostic signature (HMOX1, MAPK3, TXN) for DFU with near‐perfect accuracy (training AUC = 1, validation AUC ≥ 0.98). Multi‐omics analyses revealed significant immune dysregulation (increased B cells/CD8^+^T cells/M1 macrophages in DFS), collagen‐centric signaling dominance, and two molecular subtypes. Single‐cell RNA‐seq uncovered cell‐type‐specific dysfunction: fibroblasts and endothelial cells expanded in DFS, while HMOX1 localized to Mono‐macrophages and MAPK3 to endothelium.

**Conclusion:**

This study deeply analyzed DFU immune microenvironment characteristics, intercellular communication networks, and molecular regulatory mechanisms, revealing a dysregulated metabolic–immune repair network framework, providing new insights for understanding DFU pathological mechanisms and developing targeted diagnostic and therapeutic strategies.

## Graphical abstract

This figure comprehensively showcases the study's core findings—exceptional diagnostic performance (AUC = 1) of the taurine metabolism‐related biomarkers (HMOX1, MAPK3, TXN) in DFU, validated across independent cohorts. It integrates ROC curves, decision curve analysis (DCA), and nomograms, visually summarizing the translational potential of the research.

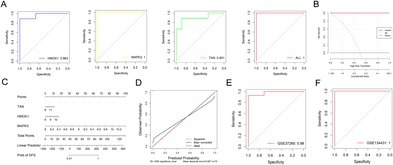

**Summary**

- This study identified HMOX1, MAPK3, and TXN as high‐accuracy diagnostic biomarkers for diabetic foot ulcers (DFU), achieving near‐perfect AUC scores (1 in training, ≥ 0.98 in validation).
- Single‐cell analysis revealed cell‐specific dysregulation, with fibroblasts and endothelial cells dominating DFU lesions, linked to oxidative stress and collagen signaling.
- The findings offer transformative potential for DFU diagnosis and targeted therapies, emphasizing metabolic–immune crosstalk as a key therapeutic avenue.

## 1 Introduction

Diabetic foot ulcers (DFU) represent a severe and prevalent complication of diabetes mellitus, posing a major global healthcare challenge [1, 2]. They are characterized by impaired wound healing within a hyperglycemic environment, arising from a complex interplay of peripheral neuropathy, peripheral arterial disease, foot deformities, and trauma [3]. Crucially, the underlying pathophysiology involves persistent inflammation, dysregulated immune responses, impaired angiogenesis, and altered cellular function [4, 5]. These factors contribute to a high risk of infection, tissue necrosis, and ultimately, amputation. This leads to significant patient morbidity, mortality, and substantial economic burden [6]. DFU is associated with high incidence rates, frequent infections, increased mortality, and frequently precedes major amputations [7]. Therefore, scientific, systematic, and individualized care for foot ulcers becomes a critical component of diabetes management. It extends far beyond simple wound treatment, encompassing a comprehensive intervention system that includes wound debridement and dressing selection, precise infection prevention and control, optimization of foot offloading strategies, vascular improvement, strict regulation of blood glucose and blood pressure, enhancement of patient education and self‐management capabilities, and multidisciplinary team collaboration [8, 9]. Effective and timely nursing interventions not only significantly promote ulcer healing and reduce amputation risk but also lay a solid foundation for patients to regain walking confidence and safeguard their foot health and freedom of movement. Despite advances in standard wound care, a significant proportion of DFUs remain refractory to treatment, highlighting the urgent need for a deeper understanding of their intricate molecular and cellular pathogenesis.

Taurine is a sulfur‐containing β‐amino acid that, although it does not participate in protein synthesis, is one of the most abundant free amino acids in the human body. It is widely distributed in the retina, myocardium, skeletal muscle, and white blood cells [10]. Taurine is generally obtained through dietary sources or synthesized de novo via the catabolism of the amino acid cysteine [11]. Taurine performs multiple functions, including scavenging reactive oxygen species (ROS) and protecting cell membranes from oxidative damage, thereby exerting antioxidant defense capabilities [12, 13]. Additionally, taurine plays a crucial role in regulating osmotic pressure and ion transport [14]. Taurine exerts beneficial effects in various diseases, such as hypertension [15], cardiovascular diseases [16], and multiple types of cancer cells [17]. Notably, taurine plays a vital role in individuals with diabetes. Studies have shown that daily supplementation of 3 g taurine safely and effectively improves metabolic and glycative stress in type 2 diabetes (T2DM) by reducing AGEs/MGO toxicity, enhancing antioxidant defenses, and improving insulin signaling [18]. Taurine also exerts a protective metabolic regulatory role in high‐fructose‐induced insulin resistance models by lowering homocysteine (Hcy) levels and alleviating oxidative stress and inflammation [19]. However, the role of taurine metabolism in DFU remains unclear. Understanding the association between taurine metabolism and DFU may aid in disease diagnosis and the development of novel metabolism‐based therapeutic approaches.

This study leveraged integrated bulk and single‐cell RNA sequencing to investigate taurine metabolism in DFU. A high‐precision diagnostic model was developed using machine learning to identify key metabolic biomarkers, demonstrating exceptional discriminatory power. Complementing this, single‐cell transcriptomic analysis systematically resolved the cellular heterogeneity within the DFU microenvironment, mapping cell‐type‐specific functions and communication networks. This combined approach provides novel insights into DFU pathogenesis and offers powerful tools for improved diagnosis and the development of targeted therapeutic strategies.

## 2 Materials and Methods

### 2.1 Data Collection

Download the microarray datasets (GSE68183 and GSE80178) for DFU from the GEO database (https://www.ncbi.nlm.nih.gov/geo/) as the training set. These datasets comprise a total of 18 samples: 9 DFU disease group samples (DFS: diabetic foot ulcer skin) and 9 control group samples (NFS: normal skin). Additionally, download the DFU microarray datasets GSE37265 and GSE134431 from GEO as the test set. GSE37265 includes 28 samples (14 DFS vs. 14 NFS), while GSE134431 includes 21 samples (13 DFS vs. 8 NFS). Further, download the single‐cell RNA sequencing dataset for DFU (GSE223964) from GEO, which contains 8 samples (5 DFS vs. 3 NFS). Finally, obtain 610 taurine metabolism‐related genes (TMRGs) from the MSigDB database (https://www.gsea‐msigdb.orggseamsigdb) (Table S1).

### 2.2 Differential Analysis and Identification of Key Modules

Differential analysis between the DFS and NFS was performed using the limma package with thresholds of adj.*p*.Val < 0.05 and |log_2_FC| > 0.585 The resulting differentially expressed genes (DEGs) were intersected with TMRGs, and the overlapping genes were submitted to the STRING database (https://www.string‐db.org/) to construct a protein–protein interaction (PPI) network using an interaction confidence score > 0.4 as the cutoff. This PPI network was imported into Cytoscape v3.9.1 (https://cytoscape.org) for visualization, and key gene modules were identified using the MCODE plugin with parameters set to degree cutoff = 2, node score cutoff = 0.2, K‐core = 2, and max depth = 100. Genes within these modules were defined as hub genes.

### 2.3 Screening Diagnostic Signature Genes to Construct a Diagnostic Model

The Machine Learning was employed to further identify key variables among the hub genes. Least absolute shrinkage and selection operator (LASSO) machine learning was performed using the “glmnet” package in R to screen for pivotal feature genes with diagnostic potential. For the critical hub genes derived from machine learning, receiver operating characteristic (ROC) curve analysis was conducted on the GSE68183 and GSE80178 datasets using the “pROC” package, where genes with AUC > 0.7 were considered diagnostically valuable. A diagnostic model was subsequently constructed based on these validated genes. Nomograms were generated using the “rmda” package, with calibration curves plotted to validate their performance. Decision curve analysis (DCA) was applied to evaluate the model's clinical utility. Finally, the model was validated in independent test sets.

### 2.4 Gene Set Enrichment Analysis and Pathway Analysis

Pathway enrichment analysis was performed on the diagnostic genes using GSEA (version 4.3.2). GO and KEGG pathway enrichment analyses were conducted on the identified DEGs employing the “clusterProfiler” R package. These analyses enabled a comprehensive assessment of enrichment patterns across biological processes, cellular components, molecular functions, and key signaling pathways.

### 2.5 Immune Infiltration Analysis

Immune infiltration scores for cell types and functional signatures were computed using ssGSEA. The CIBERSORT algorithm was utilized to further characterize differences in immune infiltration levels. Immune cell infiltration analysis was then performed using the MCP‐Counter algorithm implemented in the “IOBR” R package. Additionally, correlation analysis was performed using the corrplot package to calculate Pearson correlation coefficients both among distinct immune cell types and between the identified diagnostic signature genes and immune cells.

### 2.6 Prediction of Potential TFs (Transcription Factors) and Construction of miRNA Regulatory Networks

The miRNet database (https://www.mirnet.ca/) was employed to identify potential miRNAs targeting the diagnostic genes, with results visualized using Cytoscape. Upstream TFs were predicted via NetworkAnalyst (https://www.networkanalyst.ca/). The drug signature database DSigDB (http://dsigdb.tanlab.org/DSigDBv1.0/) was utilized to evaluate potential protein–drug interactions. Target genes were uploaded to the Enrichr suite within the gene set enrichment analysis tool (https://maayanlab.cloud/modEnrichr/) to leverage DSigDB for predicting candidate drugs that may target these genes.

### 2.7 Subtype Identification of Diagnostic Genes

The DFU disease group samples from merged datasets GSE68183, GSE80178, GSE37265, and GSE134431 were clustered using the consensus clustering algorithm in the “ConsensusClusterPlus” package based on the identified diagnostic genes. Immune infiltration heatmaps between subtypes were investigated using ssGSEA, and immune infiltration levels across subtypes were quantified via the CIBERSORT algorithm. Finally, differential analysis was performed between subtypes (thresholds: adj.*p*.Val < 0.05, |log_2_FC| > 0.585), followed by functional enrichment analysis of the resulting DEGs.

### 2.8 Processing and Annotation of Single‐Cell Data

Using the Seurat package, cell clustering was performed via UMAP and PCA analyses. Cells were filtered to exclude those with gene counts < 200 or > 2500 (nFeature_RNA) or mitochondrial gene content (mt_percent) > 5% to ensure data quality. After normalization, the FindVariableFeatures method identified 2000 highly variable genes. PCA was then applied to determine significant principal components (PCs), with 30 PCs selected for UMAP dimensionality reduction. Cells were partitioned into 17 distinct clusters using the FindClusters function at a resolution of 0.8. These clusters were annotated into 12 cell types based on canonical marker genes, and enrichment scoring for key gene sets was conducted using the AUCell package.

### 2.9 Analysis of Cell–Cell Communication

To determine the patterns of incoming and outgoing signals in cell clusters within the DFU disease group, we employed the “CellChat” package to infer and analyze intercellular communication. We used the netAnalysis_computeCentrality function to compute network centrality metrics, thereby identifying ligands and receptors in CellChat. Additionally, the netAnalysis_contribution function was utilized to calculate the contribution values of ligand–receptor pairs to the TOP1 pathway.

### 2.10 Statistical Analysis

All analyses were performed using relevant packages in the R language (version 4.4.0) and Seurat (version 4.3.0.1). The Wilcoxon test was employed to determine statistical differences between the two groups. Data visualization was primarily conducted with the “ggplot2” package. The Pearson method was used to assess associations between two variables. Statistical significance was determined at a *p* < 0.05.

## 3 Results

### 3.1 Differential Analysis of DFS and NFS

**FIGURE 1:**
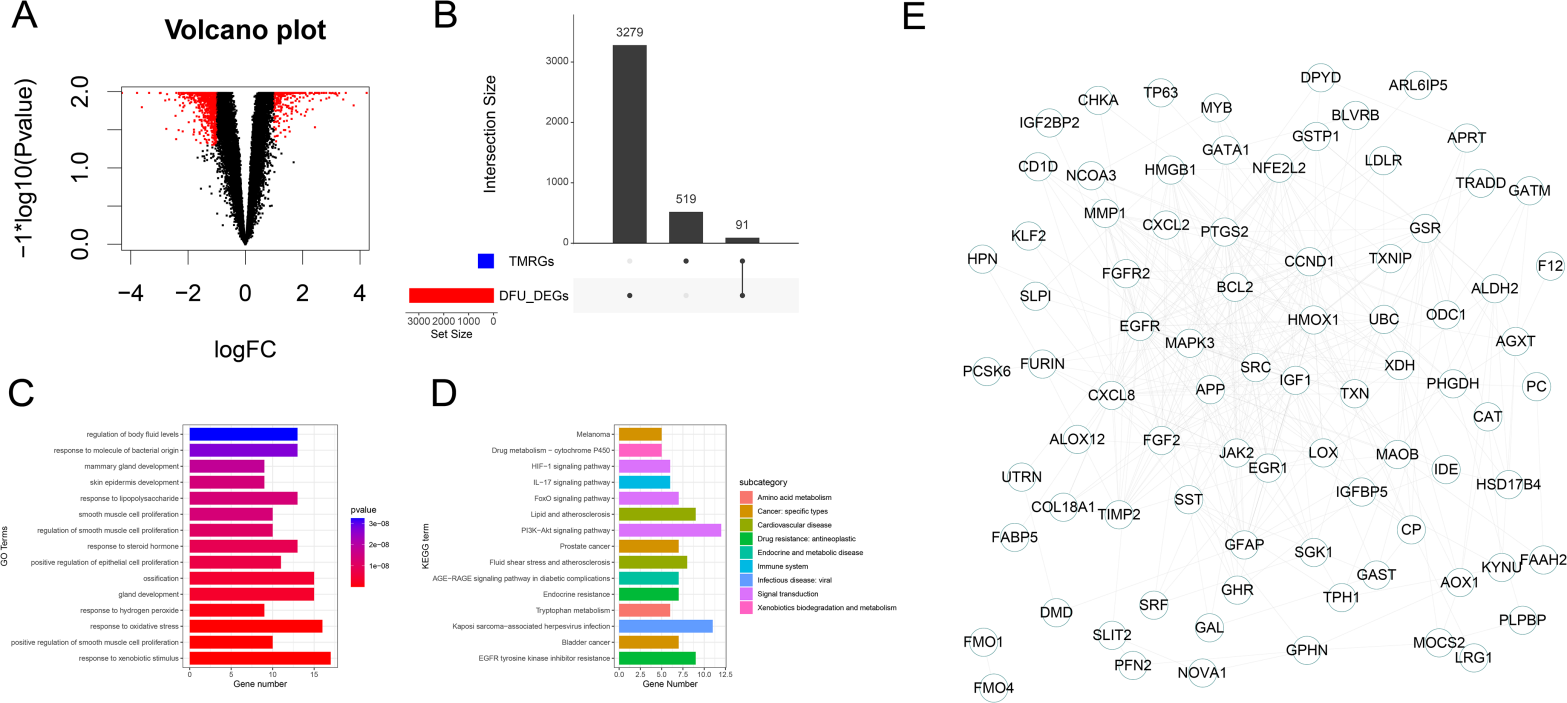
DETMRGs identification and functional analysis. (A) Volcano plot of differential analysis for DFU. (B) Intersection analysis of DEGs and TMRGs. (C) GO enrichment analysis of DETMRGs. (D) KEGG analysis of DETMRGs. (E) PPI network of DETMRGs.

Differential analysis of the training datasets (GSE68183 and GSE80178) identified 3370 DEGs, including 1402 upregulated genes and 1968 downregulated genes (Figure 1A). Intersecting these DEG with TMRGs yielded 91 differentially expressed taurine metabolism‐related genes (DETMRGs) (Figure 1B). The GO enrichment analysis reveals that these DETMRGs are primarily involved in pathways related to response to xenobiotic stimulus, response to oxidative stress, and response to steroid hormone (Figure 1C). The KEGG enrichment analysis highlights significant involvement in pathways related to the PI3K‐Akt signaling pathway, AGE‐RAGE signaling pathway in diabetic complications, and EGFR tyrosine kinase inhibitor resistance (Figure 1D). Additionally, a PPI network was constructed for the DETMRGs to elucidate interaction relationships among these genes (Figure 1E).

### 3.2 Identification of Key Modules

**FIGURE 2:**
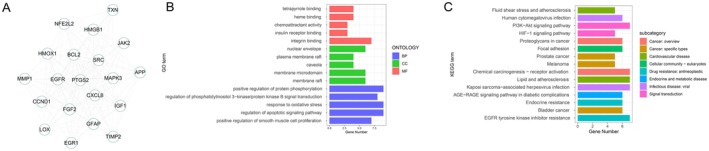
Hub genes identification and functional analysis. (A) PPI network analysis of module‐specific HUB genes. (B) GO enrichment analysis of key modules. (C) KEGG pathway analysis of key modules.

The MCODE plugin was used to score modules, ultimately screening one module defined as the key module (Table S2). Genes within the key module were identified as hub genes, with 20 hub genes detected for PPI network analysis (Figure 2A). The GO enrichment analysis of the hub genes revealed significant enrichment in positive regulation of protein phosphorylation, response to oxidative stress, and positive regulation of apoptotic signaling pathway, regulation of smooth muscle cell proliferation (Figure 2B). The KEGG enrichment analysis of the hub genes revealed significant involvement in the PI3K‐Akt signaling pathway, AGE‐RAGE signaling pathway in diabetic complications, endocrine resistance, and EGFR tyrosine kinase inhibitor resistance (Figure 2C).

### 3.3 Machine Learning‐Based Identification of Feature Genes

**FIGURE 3:**
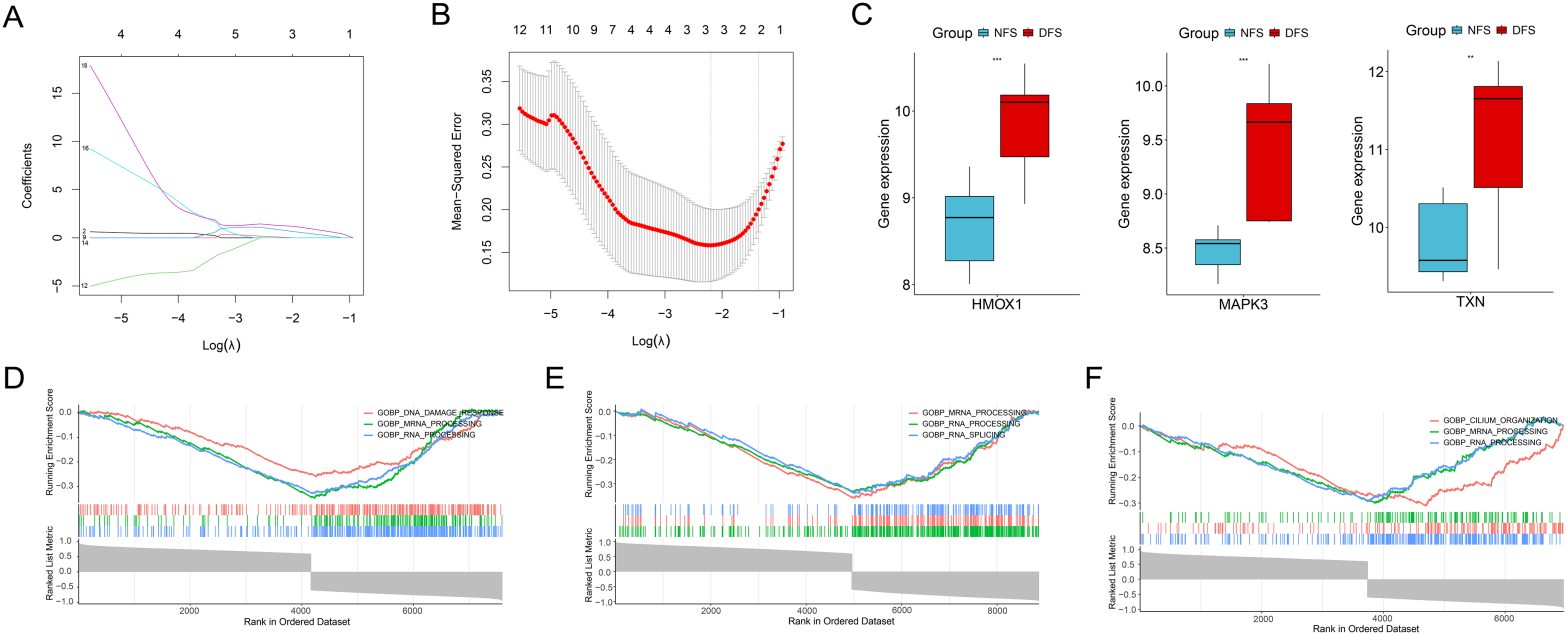
Signature gene screening. (A) Random forest importance ranking. (B) Cross‐validation curve for selecting the optimal *λ* value, and the coefficient distribution in the LASSO regression model. (C) Boxplots of three key gene expressions in NFS versus DFS. Single‐gene GSEA for HMOX1 (D), MAPK3 (E), and TXN (F).

To prevent model overfitting, LASSO regression analysis was performed on the 20 hub genes. Three key variables were identified based on the minimum lambda criterion of LASSO (Figure 3A,B). The three key genes (HMOX1 [heme oxygenase‐1], MAPK3, TXN) exhibited significantly higher expression levels in DFS compared to NFS (Figure 3C). Single‐gene GSEA revealed that HMOX1, MAPK3, and TXN were all enriched in mRNA processing and RNA processing pathways. Specifically, HMOX1 showed unique enrichment in DNA damage response, MAPK3 in RNA splicing, and TXN in cilium organization. Furthermore, the enrichment scores of all three genes exhibited negative correlations with their respective enriched pathways (Figure 3D–F).

### 3.4 Diagnostic Model Construction and Validation

**FIGURE 4:**
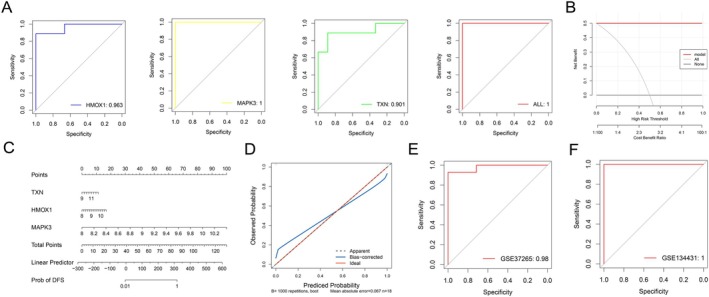
Diagnostic model development and validation. (A) ROC curve of the training set. (B) Decision curve analysis for model evaluation. (C) A nomogram was established to predict the diagnostic capability of DFU. (D) Calibration curves and clinical impact curve. ROC curve of GSE37265 (E) and GSE134431 (F).

In the training set, each individual feature gene demonstrated excellent discriminatory power, with HMOX1, MAPK3, and TXN having AUC values of 0.963 (95% CI: 0.8818–1), 1 (95% CI: 1–1), and 0.901 (95% CI: 0.7435–1), respectively (Figure 4A). The integrated model exhibited exceptional diagnostic performance with an AUC of 1 (95% CI: 1–1). DCA revealed that the predictive model consistently outperformed the default strategy across the entire risk threshold range, indicating significant clinical utility (Figure 4B). The nomogram illustrates the contributions of three genes (HMOX1, MAPK3, and TXN) to the diagnostic prediction model (Figure 4C). Additionally, calibration curves and clinical impact curves were provided to assess the nomogram's performance, demonstrating close alignment between the model's predictions and the ideal curve (Figure 4D). These robust findings were consistently replicated in the independent validation cohort: the model maintained strong predictive accuracy in the GSE37265 validation set (AUC = 0.98) (95% CI: 0.9359–1) (Figure 4E), and achieved reliable performance in the GSE134431 validation set (model AUC = 1) (95% CI: 1–1) (Figure 4F).

### 3.5 Immune Profiling Analysis of DFS and NFS

**FIGURE 5:**
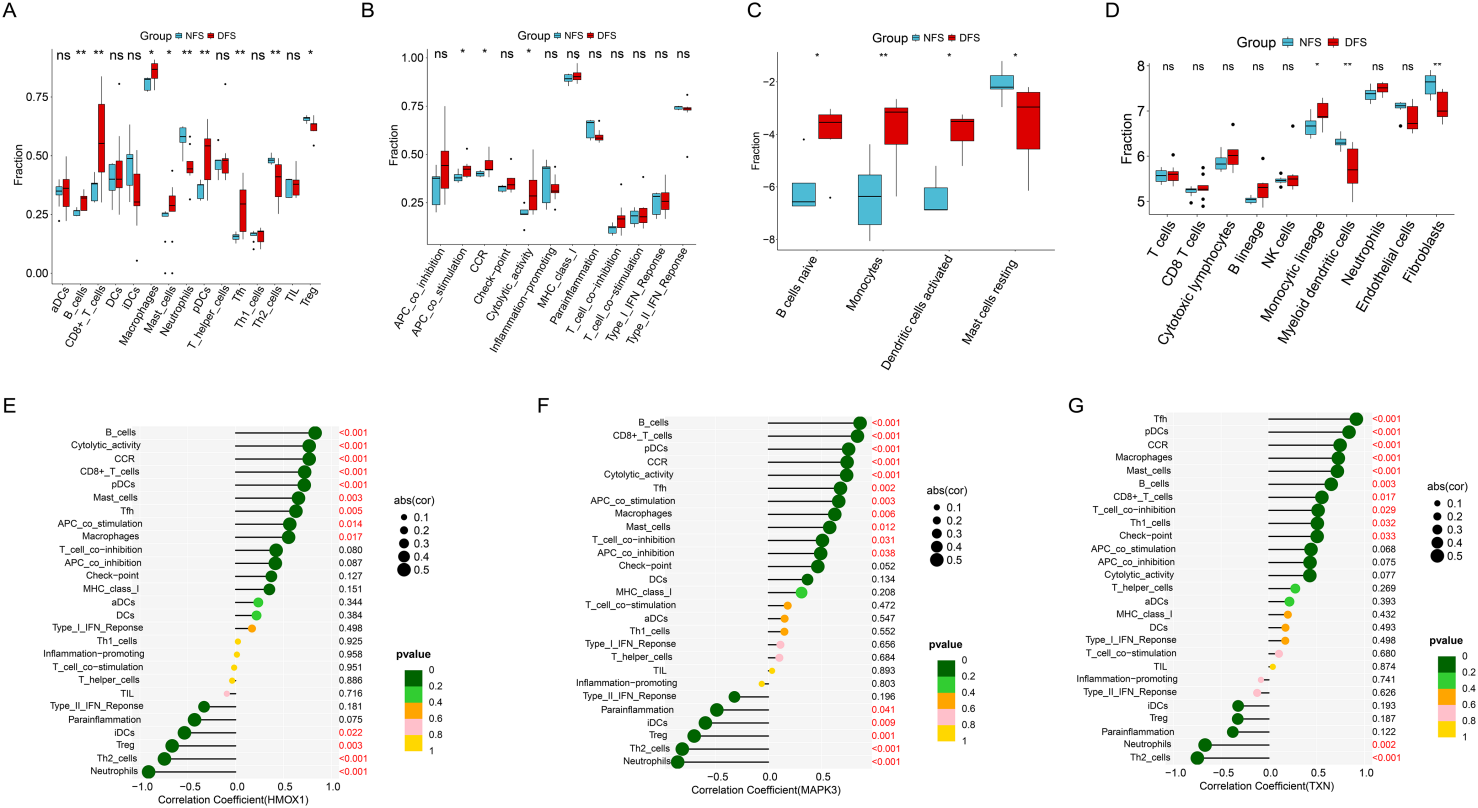
Immune microenvironment characterization. (A) ssGSEA immune cell and (B) immune function scores boxplot for NFS and DFS groups. (C) CIBERSORT immune cell fractions in NFS and DFS groups. (D) MCP‐counter box plot. Correlation between HMOX1 (E), MAPK3 (F), TXN (G), and immune infiltration levels.

Analysis using the ssGSEA algorithm revealed that DFS exhibited significantly higher levels of multiple immune cell populations compared to NFS, including B cells, CD8 + T cells, macrophages, mast cells, pDCs, and Tfh; while NFS exhibited significantly higher levels of Th2 cells and Treg (Figure 5A). Additionally, ssGSEA demonstrated elevated immune function scores in the DFS cohort (Figure 5B). CIBERSORT analysis further demonstrated significantly increased infiltration in the DFS group for the following cell subsets: naïve B cells, monocytes, and activated dendritic cells. Conversely, the NFS group exhibited elevated infiltration of resting mast cells (Figure 5C). Additionally, the scores of monocytic lineage were significantly higher in the DFS group compared to the NFS group, while myeloid dendritic cells and Fibroblasts exhibited higher scores in the NFS group (Figure 5D). All 3 genes show a strong positive correlation with B cells, CCR, pDCs, Tfh, CD8+ T cells, macrophages, and mast cells, while they exhibit a strong negative correlation with neutrophils and Th2 cells (Figure 5E–G).

### 3.6 Construction of Potential TFs and miRNA Regulatory Networks and Therapeutic Targeting Prediction

**FIGURE 6:**
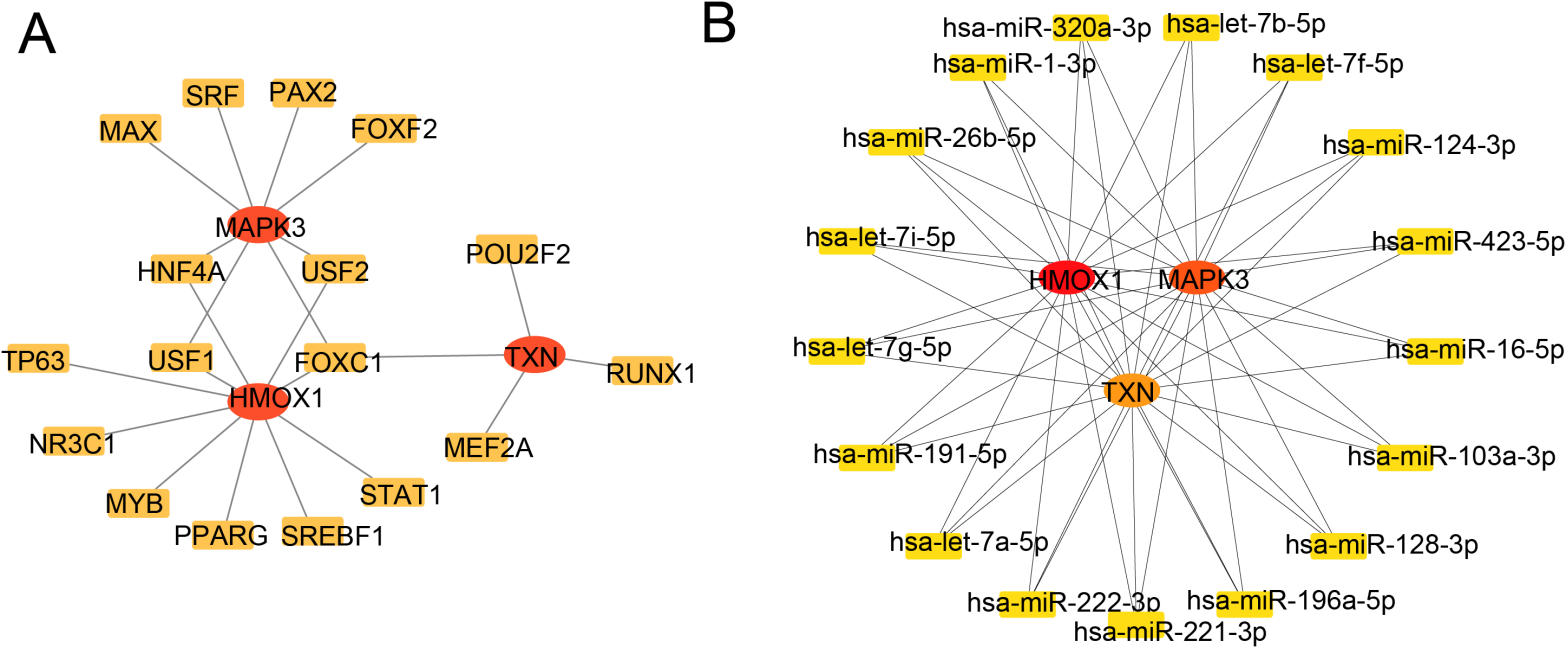
Construction of regulatory networks based on signature genes. (A) TF‐gene interaction network. (B) miRNA network of signature genes.

The three target genes (HMOX1, MAPK3, TXN) are regulated by complex transcriptional networks. Multiple transcription factors, including HNF4A, USF1, USF2, and FOXC1, were identified as key regulators interacting with several diagnostic genes, suggesting their central role in transcriptional regulation (Figure 6A). Within the miRNA regulatory network, the three target genes are subject to intricate miRNA‐mediated control, with specific miRNAs such as hsa‐miR‐1‐3p and hsa‐miR‐128‐3p demonstrating extensive regulatory interactions with these target genes (Figure 6B). Based on predictive results from the DSigDB database, hypochlorous acid, L‐cystathionine, buthionine sulfoximine, tert‐butyl hydroperoxide, and acrolein were identified as potential therapeutic agents targeting the three diagnostic signature genes (Tables 1 and S3).

**TABLE 1:** Drug prediction for diagnostic genes.

Term	*p*	Adjusted *p*	Odds ratio	Combined score	Genes
Hypochlorous acid BOSS	1.15E−09	1.19E−06	59 934	1 233 395	HMOX1; TXN; MAPK3
l‐cystathionine BOSS	2.19E−09	1.19E−06	59 919	1 194 647	HMOX1; TXN; MAPK3
Buthionine sulfoximine BOSS	3.04E−09	1.19E−06	59 910	1 174 823	HMOX1; TXN; MAPK3
Tert‐butyl hydroperoxide BOSS	4.09E−09	1.20E−06	59 901	1 156 943	HMOX1; TXN; MAPK3
Acrolein BOSS	5.35E−09	1.26E−06	59 892	1 140 659	HMOX1; TXN; MAPK3

### 3.7 Molecular Subtyping Based on DETMRGs

**FIGURE 7:**
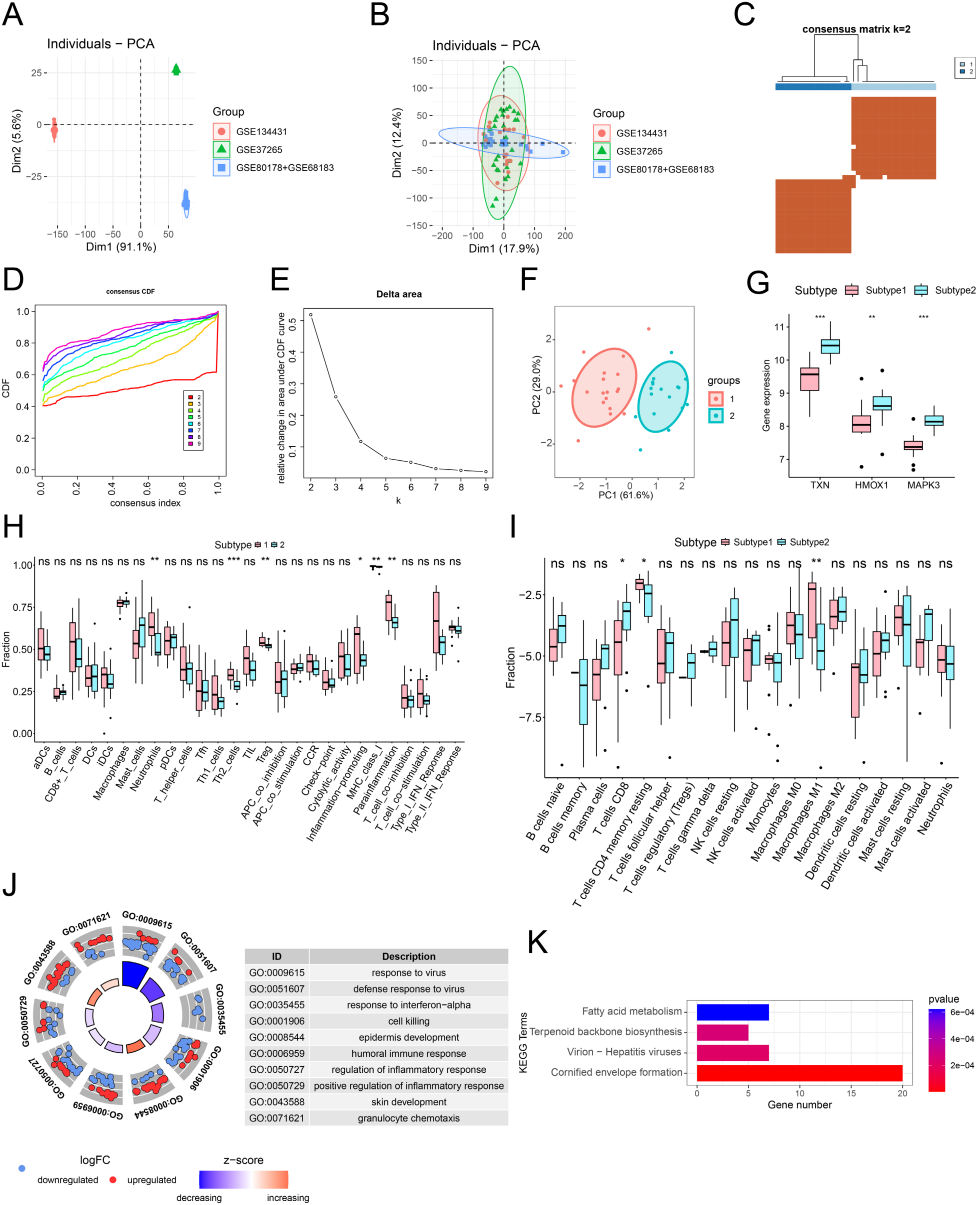
Cluster identification and immune profiling. (A) PCA analysis prior to batch effect removal. (B) PCA analysis prior to batch effect removal. (C) Consensus clustering heatmap based on prognosis‐related DETMRGs. (D) Cumulative distribution function plot for cluster stability. (E) Relative change in area under CDF curve. (F) Inter‐subtype PCA analysis. (G) Expression profiles of three candidate genes across different subtypes. (H) ssGSEA immune cell for different subtypes. (I) CIBERSORT immune cell fractions in different subtypes. GO (J) and KEGG (K) enrichment analyses of inter‐subtype DEGs.

To facilitate robust integration of DFU disease group samples from datasets GSE68183, GSE80178, GSE37265, and GSE134431, batch effect correction was performed across these four datasets (Figure 7A,B). Based on three diagnostic‐related DETMRG datasets, consensus clustering analysis was conducted using the “ConsensusClusterPlus” package, with the optimal cluster number determined as *K* = 2 (Figure 7C–E). PCA analysis visually demonstrated distinct separation between subtype 1 and subtype 2 (Figure 7F). The expression of the three diagnostic genes was significantly higher in subtype 2 than in subtype 1 (Figure 7G). ssGSEA demonstrated that both immune cell scores and immune infiltration subtype scores were significantly higher in subtype 1 compared to subtype 2 (Figure 7H). CIBERSORT analysis further demonstrated significantly increased infiltration in subtype 2 for the following cell subsets: T cells CD8 while subtype 1 exhibited elevated infiltration of T cells CD4 memory resting and macrophages M1 (Figure 7I). Additionally, enrichment analysis of inter‐subtype differences genes revealed that DEGs were primarily enriched in the following terms: response to virus, defense response to virus, response to interferon‐alpha, and cell killing, as demonstrated by GO enrichment analysis (Figure 7J). KEGG analysis revealed that the DEGs were predominantly enriched in the cornified envelope formation pathway (Figure 7K).

### 3.8 Processing and Annotation of Single‐Cell Data

**FIGURE 8:**
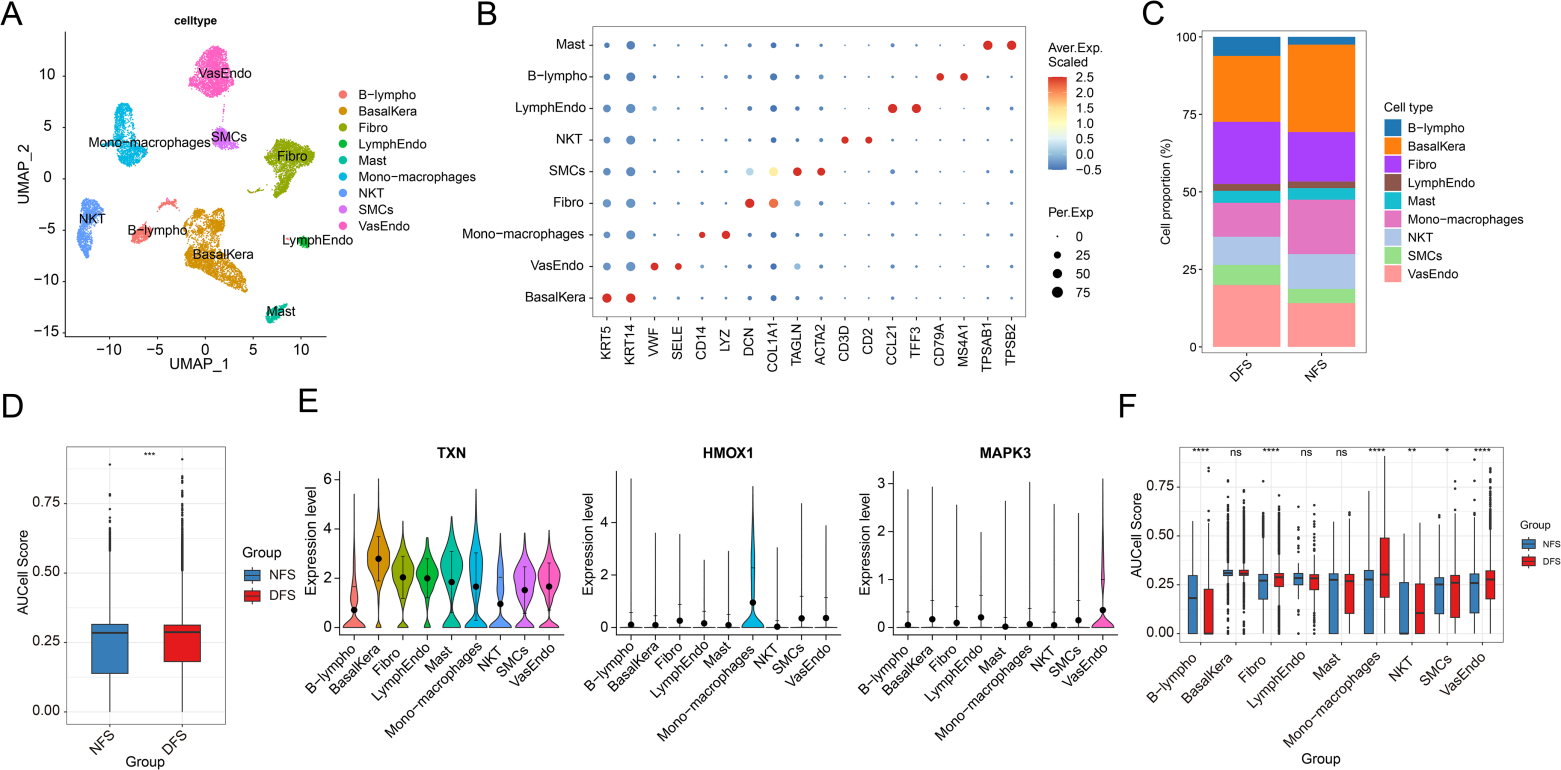
Single‐cell classification and annotation. (A) UMAP plot showing cell subpopulations in DFU. (B) Bubble heatmap of expression levels for selected marker genes in DFU. (C) Bar plot of cell proportions between DFS and NFS groups. (D) Violin plots of diagnostic genes across distinct cell clusters. (E) Box plot of AUCell score between DFS and NFS groups. (F) Box plots of AUCell score for DFS and NFS groups across different cell clusters.

Following integration and processing of the single‐cell data, a total of 14 498 cells were obtained. Using canonical marker genes, these cells were annotated into 9 biologically defined cell types: smooth muscle cells (SMCs: TAGLN, ACTA2), fibroblasts (Fibro: DCN, COL1A1), mono‐macrophages (Mono‐macrophages: CD14, LYZ), vascular endothelial cells (VasEndo: VWF, SELE), basal keratinocytes (BasalKera: KRT5, KRT14), NK and T cells (NKT: CD3D, CD2), lymphatic endothelial cells (LymphEndo: CCL21, TFF3), B lymphocytes (B‐lympho: CD79A, MS4A1), and mast cells (Mast: TPSAB1, TPSB2) (Figure 8A,B). Comparative analysis of cellular composition revealed significant differences between the NFS and DFS groups. The NFS group exhibited significantly higher proportions of BasalKera and Mono‐macrophages compared to the DFS group. Conversely, the DFS cohort demonstrated elevated fractions of VasEndo and Fibro (Figure 8C). Additionally, TXN was expressed across all cell types, while HMOX1 expression was exclusively restricted to Mono‐macrophages, and MAPK3 was detected solely in VasEndo (Figure 8D). Comparative analysis of AUCell scores revealed that the DFS group exhibited significantly higher AUCell scores than the NFS group (Figure 8E). Additionally, it was found that the DFS group exhibited significantly higher scores than the NFS group in Fibro, Mono‐macrophages, NKT, SMCs, and VasEndo (Figure 8F).

### 3.9 Cell Communication Analysis

**FIGURE 9:**
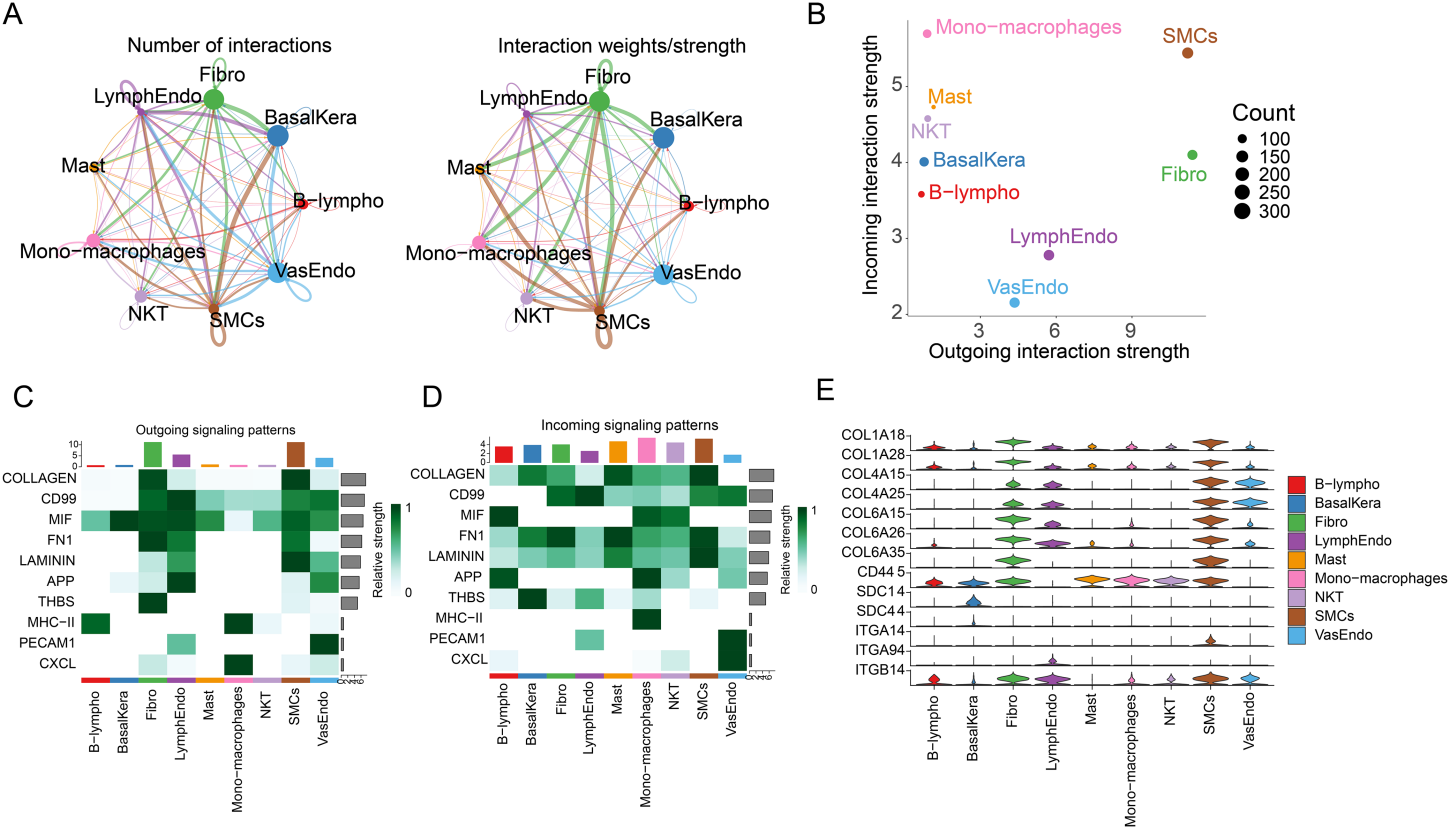
Cell–cell signaling communication analysis. (A) CellChat circular plot of the DFS group. (B) CellChat scatter plot of the DFS group. (C) Contribution of each cell cluster to outgoing signaling pathways in DFS. (D) Contribution of each cell cluster to incoming signaling pathways in DFS. (E) Heatmap of expression levels for all genes in the COLLAGEN pathway across cell clusters.

Cell communication between different cell types is highly active and complex, potentially involving the coordination or regulation of multiple signaling pathways. Fibro, SMCs, VasEndo, and BasalKera serve as core hubs in the cellular communication network, participating in the highest number of interactions (sending and receiving the most signals) and exhibiting exceptionally strong interaction strength/weight with other cell types (Figure 9A). SMCs and Fibro show high outgoing interaction strength, while Mono‐macrophages exhibit high incoming interaction strength (Figure 9B). BasalKera exhibit high‐intensity outgoing signals in the MIF signaling pathway, while Mono‐macrophages show high‐intensity outgoing signals in the CXCL signaling pathway. Fibro, LymphEndo, SMCs, and VasEndo demonstrate high outgoing signal strength across multiple signaling pathways (Figure 9C). Nine distinct cell types all exhibit high signal reception strength across multiple signaling pathways (Figure 9D). Additionally, within the COLLAGEN signaling network, it was discovered that COL1A18, COL1A28, COL6A15, COL6A26, and COL6A35 exhibit significant contribution values in Fibro and SMCs; CD445 and ITGB14 show substantial contribution values across multiple cell types; while COL4A15 and COL4A25 demonstrate significant contribution values in SMCs and VasEndo (Figure 9E).

## 4 Discussion

This study presents a comprehensive multi‐omics investigation into the role of taurine metabolism in DFU, integrating bulk RNA sequencing, single‐cell transcriptomics, and computational systems biology approaches. By systematically analyzing molecular dysregulation across cellular and tissue‐level landscapes, we aimed to unravel the interplay between taurine metabolism, immune dynamics, and collagen‐centric cellular crosstalk in DFU pathogenesis. These findings collectively highlight the potential of taurine metabolism as a pivotal axis in DFU pathophysiology, bridging metabolic dysfunction, inflammatory responses, and tissue repair processes, while offering novel insights for biomarker discovery and targeted therapeutic strategies.

The identification of HMOX1, MAPK3, and TXN as robust diagnostic biomarkers underscores their pivotal roles in DFU pathogenesis. HMOX1 is a critical antioxidant enzyme that mitigates oxidative stress by degrading heme into biliverdin, carbon monoxide, and free iron [20, 21]. HMOX1 is significantly upregulated in multiple diseases, such as proliferative diabetic retinopathy and diabetic cardiomyopathy [22, 23], where it plays a critical role in regulating iron homeostasis and providing cytoprotection. In this study, the upregulation of HMOX1 in the DFS group may represent a compensatory mechanism: sustained hyperglycemia exacerbates oxidative damage, and elevated HMOX1 expression protects cells against ROS‐induced injury. MAPK3 (extracellular signal‐regulated kinase 1, ERK1) regulates cell proliferation, differentiation, and inflammatory responses [24, 25]. Research on MAPK3 in diabetes and its complications has yielded multiple findings. Studies have discovered that specific polyphenols (such as quercetin and kaempferol) in whole green jackfruit powder can improve insulin resistance and lipid metabolism disorders by inhibiting the overactivation of MAPK3, providing a novel target for dietary intervention in obesity‐associated T2DM [26]. Research has demonstrated that MAPK3 is highly expressed in patients with diabetic foot, and its overactivation promotes the expression of matrix metalloproteinase 9. This leads to dysregulation of extracellular matrix degradation, thereby delaying the healing of DFU wounds [27]. Consistent with these findings, this study also revealed high expression of MAPK3 in DFS. TXN (thioredoxin) maintains cellular redox homeostasis by scavenging ROS and regulating apoptosis [28]. TXN, as a key regulator of oxidative stress, is increasingly recognized for its role in diabetes and its complications. Studies have found that overexpression of TXN can lower blood glucose levels in diabetic model animals, counteract oxidative stress, and improve β‐cell function [29]. Based on the collective evidence, HMOX1, MAPK3, and TXN form an interconnected triad that synergistically drives DFU pathogenesis through oxidative stress amplification, impaired matrix remodeling, and dysregulated cellular repair pathways, thereby establishing them as pivotal therapeutic targets for diabetic wound healing intervention.

Besides, in different genes, taurine exhibits taurine‐specific mechanisms. The relationship between HMOX1 and taurine metabolism is not simply linear; rather, they are dynamically coupled through the oxidative stress–antioxidant network with Nrf2 as a central node, collectively maintaining cellular redox homeostasis. Taurine can directly scavenge ROS, thus affecting the expression demand for HMOX1 [30, 31]. Concurrently, it can indirectly upregulate HMOX1 through Nrf2, creating a synergistic effect [32]. Studies have shown that taurine promotes insulin synthesis through a complex signaling pathway involving miR‐7a/RAF1/ERK1/2, demonstrating that taurine can modulate the activity of the MAPK3 pathway, thereby improving insulin‐related functions [33].

Our research indicates significant enrichment of the PI3K‐Akt signaling pathway, and the AGE‐RAGE signaling pathway in DFU. Studies have demonstrated that activation of the PI3K‐Akt/mTOR pathway upregulates the expression of growth factors such as VEGF, FGF, and EGF, promoting the proliferation, migration, and collagen synthesis of keratinocytes, fibroblasts, and endothelial cells, thereby accelerating DFU wound closure [3]. Additional research has shown that taurine significantly reduces ROS levels in diabetic wounds, inhibits oxidative stress, and shortens diabetic wound healing time by regulating the PI3K‐Akt‐mTOR axis to reduce inflammatory cytokines and increase anti‐inflammatory and growth factor secretion [34]. Additionally, studies have confirmed that blocking the AGE‐RAGE axis (using soluble RAGE decoys, RAGE‐neutralizing antibodies, or natural compounds like quercetin) significantly reduces local inflammation, improves collagen remodeling, and accelerates DFU closure [35].

The single‐cell sequencing results reveal distinct pathological features of microenvironmental dysregulation across different cellular subpopulations in wound healing. Specifically, the elevated proportions of Fibro and VasEndo in DFS indicate an active reparative state at the wound site. Studies demonstrate that under hyperglycemic conditions, fibroblasts differentiate into α‐SMA^+^ myofibroblasts, which secrete collagen and fibronectin while facilitating wound contraction [36]. Meanwhile, vascular endothelial cells maintain microvascular tone through eNOS‐mediated nitric oxide production and recruit endothelial progenitor cells (EPCs) to drive neovascularization [37]. Notably, the exclusive expression of HMOX1 in Mono‐macrophages highlights its specific niche role in regulating macrophage‐mediated oxidative stress responses [38, 39]. Conversely, MAPK3 detection restricted to vascular endothelial cells implicates its involvement in monocyte recruitment and endothelial dysfunction [40]. In contrast, the ubiquitous expression of TXN across all cell types reflects a systemic adaptation to chronic oxidative stress in DFU. Furthermore, the immune microenvironment in DFU exhibits significant imbalance, characterized by the coexisting hyperactivation of pro‐inflammatory immunity and impaired immunoregulatory function. The DFS group shows markedly increased infiltration of B cells, CD8^+^ T cells, macrophages, and mast cells. The strong positive correlations between immune‐related genes (HMOX1, MAPK3, TXN) and these immune subpopulations underscore their critical roles in modulating inflammatory cascades.

Integrating multi‐omics research findings into DFU treatment strategies holds significant promise for improving clinical outcomes. Our study identifies oxidative stress and collagen‐centric signaling as core drivers of DFU pathology, suggesting that combining antioxidant therapies (e.g., topical N‐acetylcysteine or taurine supplementation) with conventional wound care may synergistically mitigate redox imbalance as an innovative nursing intervention. Despite the study finding a correlation between the diagnostic genes and immune cells and linking them to taurine metabolism, it did not validate the direct mechanistic regulatory relationships. Further research is needed to verify the specific roles of these genes within the metabolic‐immune repair network. Additionally, the study relies exclusively on publicly available transcriptomic data (bulk RNA‐seq and scRNA‐seq) from the GEO database. While this approach is effective for identifying potential biomarkers, it lacks validation with an in‐house patient cohort. This may limit the generalizability of the findings to other populations. Future DFU care models should adopt multidisciplinary strategies integrating advanced diagnostics, targeted biologics, and mechanism‐based dressings to disrupt the vicious cycle of inflammation, oxidative damage, and impaired repair—ultimately.

## 5 Conclusion

This study conducted an in‐depth analysis of the immune microenvironment characteristics, intercellular communication networks, and molecular regulatory mechanisms in DFU, revealing a dysregulated metabolic–immune repair network framework. This work offers new perspectives on the pathological mechanisms of DFU. It may also provide a theoretical basis for developing targeted diagnostic and therapeutic strategies.

## Author Contributions

M.H. and J.C. contributed to the study design. M.H. and J.C. conducted the literature search. M.H. and J.C. acquired the data. J.C. wrote the article and performed data analysis. M.H. revised the article and gave the final approval of the version to be submitted. All authors read and approved the final manuscript.

## Ethics Statement

The authors have nothing to report.

## Consent

The authors have nothing to report.

## Conflicts of Interest

The authors declare no conflicts of interest.

## Supporting information


**Table S1:** Summation of TMRGs.
**Table S2:** MCODE scoring results for modules.
**Table S3:** Drug prediction results (DSigDB).

## Data Availability

The data and materials in the current study are available from the corresponding author on reasonable request.

## References

[1] L. Yang , G. C. Rong , and Q. N. Wu , “Diabetic Foot Ulcer: Challenges and Future,” World Journal of Diabetes 13, no. 12 (2022): 1014–1034. 36578870 10.4239/wjd.v13.i12.1014 PMC9791573

[2] J. M. Raja , M. A. Maturana , S. Kayali , A. Khouzam , and N. Efeovbokhan , “Diabetic Foot Ulcer: A Comprehensive Review of Pathophysiology and Management Modalities,” World Journal of Clinical Cases 11, no. 8 (2023): 1684–1693. 36970004 10.12998/wjcc.v11.i8.1684 PMC10037283

[3] H. Deng , B. Li , Q. Shen , et al., “Mechanisms of Diabetic Foot Ulceration: A Review,” Journal of Diabetes 15, no. 4 (2023): 299–312. 36891783 10.1111/1753-0407.13372 PMC10101842

[4] D. Zhu , W. Wei , J. Zhang , B. Zhao , Q. Li , and P. Jin , “Mechanism of Damage of HIF‐1 Signaling in Chronic Diabetic Foot Ulcers and Its Related Therapeutic Perspectives,” Heliyon 10, no. 3 (2024): e24656. 38318060 10.1016/j.heliyon.2024.e24656 PMC10839564

[5] A. S. Raja Asunama , N. Nambi , L. Radhakrishnan , M. K. Prasad , and K. M. Ramkumar , “Neutrophil Migration Is a Crucial Factor in Wound Healing and the Pathogenesis of Diabetic Foot Ulcers: Insights Into Pharmacological Interventions,” Current Vascular Pharmacology 23, no. 2 (2024): 98–112. 10.2174/0115701611308960241014155413 39482919

[6] U. Riedel , E. Schüßler , D. Härtel , A. Keiler , S. Nestoris , and H. Stege , “Wound Treatment in Diabetes Patients and Diabetic Foot Ulcers,” Hautarzt 71, no. 11 (2020): 835–842. 33044558 10.1007/s00105-020-04699-9

[7] C. W. Hicks , J. K. Canner , N. Mathioudakis , C. Lippincott , R. L. Sherman , and C. J. Abularrage , “Incidence and Risk Factors Associated With Ulcer Recurrence Among Patients With Diabetic Foot Ulcers Treated in a Multidisciplinary Setting,” Journal of Surgical Research 246 (2020): 243–250. 31610352 10.1016/j.jss.2019.09.025

[8] K. Parveen , M. A. Hussain , S. Anwar , H. M. Elagib , and M. A. Kausar , “Comprehensive Review on Diabetic Foot Ulcers and Neuropathy: Treatment, Prevention and Management,” World Journal of Diabetes 16, no. 3 (2025): 100329. 40093290 10.4239/wjd.v16.i3.100329 PMC11885961

[9] X. Wang , C. X. Yuan , B. Xu , and Z. Yu , “Diabetic Foot Ulcers: Classification, Risk Factors and Management,” World Journal of Diabetes 13, no. 12 (2022): 1049–1065. 36578871 10.4239/wjd.v13.i12.1049 PMC9791567

[10] Z. Feng , Y. Yang , W. Luo , et al., “Integrative Analysis of Taurine Metabolism‐Related Genes Prognostic Signature With Immunotherapy and Identification of ABCB1 and GORASP1 as Key Genes in Nasopharyngeal Carcinoma,” Amino Acids 57, no. 1 (2025): 21. 40272558 10.1007/s00726-025-03452-7 PMC12021963

[11] K. Duszka , “Versatile Triad Alliance: Bile Acid, Taurine and Microbiota,” Cells 11, no. 15 (2022): 2337. 35954180 10.3390/cells11152337 PMC9367564

[12] C. J. Jong , P. Sandal , and S. W. Schaffer , “The Role of Taurine in Mitochondria Health: More Than Just an Antioxidant,” Molecules 26, no. 16 (2021): 4913. 34443494 10.3390/molecules26164913 PMC8400259

[13] S. Baliou , M. Adamaki , P. Ioannou , et al., “Protective Role of Taurine Against Oxidative Stress (Review),” Molecular Medicine Reports 24, no. 2 (2021): 605. 34184084 10.3892/mmr.2021.12242 PMC8240184

[14] Y. Zhou , S. Pei , G. Qiu , et al., “Taurine Is Essential for Mouse Uterine Luminal Fluid Resorption During Implantation Window via the SCNN1A and AQP8 Signaling†,” Biology of Reproduction 112, no. 1 (2025): 140–155. 39428112 10.1093/biolre/ioae152

[15] C. C. Tzang , W. C. Lin , L. H. Lin , et al., “Insights Into the Cardiovascular Benefits of Taurine: A Systematic Review and Meta‐Analysis,” Nutrition Journal 23, no. 1 (2024): 93. 39148075 10.1186/s12937-024-00995-5 PMC11325608

[16] G. Santulli , U. Kansakar , F. Varzideh , P. Mone , S. S. Jankauskas , and A. Lombardi , “Functional Role of Taurine in Aging and Cardiovascular Health: An Updated Overview,” Nutrients 15, no. 19 (2023): 4236. 37836520 10.3390/nu15194236 PMC10574552

[17] S. Tu , X. L. Zhang , H. F. Wan , et al., “Effect of Taurine on Cell Proliferation and Apoptosis Human Lung Cancer A549 Cells,” Oncology Letters 15, no. 4 (2018): 5473–5480. 29552188 10.3892/ol.2018.8036 PMC5840730

[18] F. Esmaeili , V. Maleki , S. Kheirouri , and M. Alizadeh , “The Effects of Taurine Supplementation on Metabolic Profiles, Pentosidine, Soluble Receptor of Advanced Glycation End Products and Methylglyoxal in Adults With Type 2 Diabetes: A Randomized, Double‐Blind, Placebo‐Controlled Trial,” Canadian Journal of Diabetes 45, no. 1 (2021): 39–46. 32861603 10.1016/j.jcjd.2020.05.004

[19] H. O. El Mesallamy , E. El‐Demerdash , L. N. Hammad , and H. M. El Magdoub , “Effect of Taurine Supplementation on Hyperhomocysteinemia and Markers of Oxidative Stress in High Fructose Diet Induced Insulin Resistance,” Diabetology & Metabolic Syndrome 2 (2010): 46. 20591133 10.1186/1758-5996-2-46 PMC2907312

[20] W. Lv , S. Hu , F. Yang , et al., “Heme Oxygenase‐1: Potential Therapeutic Targets for Periodontitis,” PeerJ 12 (2024): e18237. 39430558 10.7717/peerj.18237 PMC11488498

[21] J. J. Lu , A. Abudukeyoumu , X. Zhang , L. B. Liu , M. Q. Li , and F. Xie , “Heme Oxygenase 1: A Novel Oncogene in Multiple Gynecological Cancers,” International Journal of Biological Sciences 17, no. 9 (2021): 2252–2261. 34239353 10.7150/ijbs.61073 PMC8241721

[22] J. Zhen , X. Sheng , T. Chen , and H. Yu , “Histone Acetyltransferase Kat2a Regulates Ferroptosis via Enhancing Tfrc and Hmox1 Expression in Diabetic Cardiomyopathy,” Cell Death & Disease 15, no. 6 (2024): 406. 38858351 10.1038/s41419-024-06771-x PMC11164963

[23] H. Zhou , L. Zhang , C. Ding , Y. Zhou , and Y. Li , “Upregulation of HMOX1 Associated With M2 Macrophage Infiltration and Ferroptosis in Proliferative Diabetic Retinopathy,” International Immunopharmacology 134 (2024): 112231. 38739977 10.1016/j.intimp.2024.112231

[24] A. Taherkhani , P. Khodadadi , L. Samie , Z. Azadian , and Z. Bayat , “Flavonoids as Strong Inhibitors of MAPK3: A Computational Drug Discovery Approach,” International Journal of Analytical Chemistry 2023 (2023): 8899240. 37090055 10.1155/2023/8899240 PMC10121358

[25] A. Martin‐Vega and M. H. Cobb , “ERK1/2‐MAPK Signaling: Metabolic, Organellar, and Cytoskeletal Interactions,” Current Opinion in Cell Biology 95 (2025): 102526. 40344863 10.1016/j.ceb.2025.102526

[26] T. Maradesha , R. M. Martiz , S. M. Patil , et al., “Integrated Network Pharmacology and Molecular Modeling Approach for the Discovery of Novel Potential MAPK3 Inhibitors From Whole Green Jackfruit Flour Targeting Obesity‐Linked Diabetes Mellitus,” PLoS One 18, no. 1 (2023): e0280847. 36716329 10.1371/journal.pone.0280847 PMC9886246

[27] X. Wang , G. Jiang , J. Zong , et al., “Revealing the Novel Ferroptosis‐Related Therapeutic Targets for Diabetic Foot Ulcer Based on the Machine Learning,” Frontiers in Genetics 13 (2022): 944425. 36226171 10.3389/fgene.2022.944425 PMC9549267

[28] J. Zhang , X. Li , Z. Zhao , W. Cai , and J. Fang , “Thioredoxin Signaling Pathways in Cancer,” Antioxidants & Redox Signaling 38, no. 4–6 (2023): 403–424. 35686449 10.1089/ars.2022.0074

[29] I. Kokkinopoulou and A. Papadopoulou , “Thioredoxin‐Interacting Protein (TXNIP) in Gestational Diabetes Mellitus,” Metabolites 15, no. 6 (2025): 351. 40559375 10.3390/metabo15060351 PMC12195283

[30] D. S. Lee and S. H. Cheong , “Taurine Have Neuroprotective Activity Against Oxidative Damage‐Induced HT22 Cell Death Through Heme Oxygenase‐1 Pathway,” Advances in Experimental Medicine and Biology 975, no. 1 (2017): 159–171. 28849452 10.1007/978-94-024-1079-2_14

[31] U. Seidel , K. Lüersen , P. Huebbe , and G. Rimbach , “Taurine Enhances Iron‐Related Proteins and Reduces Lipid Peroxidation in Differentiated C2C12 Myotubes,” Antioxidants 9, no. 11 (2020): 1071. 33142756 10.3390/antiox9111071 PMC7693586

[32] S. I. Seol , I. S. Kang , J. S. Lee , J. K. Lee , and C. Kim , “Taurine Chloramine‐Mediated Nrf2 Activation and HO‐1 Induction Confer Protective Effects in Astrocytes,” Antioxidants 13, no. 2 (2024): 169. 38397767 10.3390/antiox13020169 PMC10886344

[33] H. Liu , T. Niu , G. Qiu , S. Cui , and D. Zhang , “Taurine Promotes Insulin Synthesis by Enhancing Isl‐1 Expression Through miR‐7a/RAF1/ERK1/2 Pathway,” In Vitro Cellular & Developmental Biology—Animal 60, no. 1 (2024): 23–35. 38117455 10.1007/s11626-023-00835-6

[34] Z. Luo , S. Zheng , Z. Hu , et al., “Ultrasound‐Responsive Taurine Lipid Nanoparticles Attenuate Oxidative Stress and Promote Macrophage Polarization for Diabetic Wound Healing,” Free Radical Biology & Medicine 233 (2025): 302–316. 40187503 10.1016/j.freeradbiomed.2025.04.007

[35] M. Radziszewski , R. Galus , K. Łuszczyński , et al., “The RAGE Pathway in Skin Pathology Development: A Comprehensive Review of Its Role and Therapeutic Potential,” International Journal of Molecular Sciences 25, no. 24 (2024): 13570. 39769332 10.3390/ijms252413570 PMC11676465

[36] A. H. A. Al‐Rikabi , D. J. Tobin , K. Riches‐Suman , and M. J. Thornton , “Dermal Fibroblasts Cultured From Donors With Type 2 Diabetes Mellitus Retain an Epigenetic Memory Associated With Poor Wound Healing Responses,” Scientific Reports 11, no. 1 (2021): 1474. 33446687 10.1038/s41598-020-80072-z PMC7809350

[37] S. C. Selvakumar , K. A. Preethi , and D. Sekar , “MicroRNA‐510‐3p Regulated Vascular Dysfunction in Preeclampsia by Targeting Vascular Endothelial Growth Factor A (VEGFA) and Its Signaling Axis,” Placenta 153 (2024): 31–52. 38820941 10.1016/j.placenta.2024.05.135

[38] W. Chen , Y. Zhang , J. Chen , et al., “Heme Oxygenase‐1 Modulates Macrophage Polarization Through Endothelial Exosomal miR‐184‐3p and Reduces Sepsis‐Induce Lung Injury,” International Journal of Nanomedicine 20 (2025): 5039–5057. 40264818 10.2147/IJN.S506830 PMC12013636

[39] L. Hu , Y. Yu , Y. Shen , et al., “Ythdf2 Promotes Pulmonary Hypertension by Suppressing Hmox1‐Dependent Anti‐Inflammatory and Antioxidant Function in Alveolar Macrophages,” Redox Biology 61 (2023): 102638. 36801705 10.1016/j.redox.2023.102638 PMC9975317

[40] Y. Feng , S. Q. Tu , Y. L. Hou , et al., “Alendronate Sodium Induces G1 Phase Arrest and Apoptosis in Human Umbilical Vein Endothelial Cells by Inhibiting ROS‐Mediated ERK1/2 Signaling,” Toxicology 508 (2024): 153917. 39137827 10.1016/j.tox.2024.153917

